# Airway models in a pandemic: Suitability of models in modeling SARS-CoV-2

**DOI:** 10.1371/journal.ppat.1010432

**Published:** 2022-03-29

**Authors:** Andrew Teo, Caroline Lin Lin Chua, Louisa L. Y. Chan

**Affiliations:** 1 Lee Kong Chian School of Medicine, Nanyang Technological University, Singapore, Singapore; 2 Department of Medicine, The Doherty Institute, University of Melbourne, Melbourne, Australia; 3 School of Biosciences, Faculty of Health and Medicine Sciences, Taylor’s University, Subang Jaya, Malaysia; University of Wisconsin-Madison, UNITED STATES

In a global pandemic involving respiratory pathogens such as Severe Acute Respiratory Syndrome Coronavirus 2 (SARS-CoV-2), intensified scientific research is required to delineate pathways involved in infectivity, transmissibility, and pathogenicity of the causative pathogen. SARS-CoV-2, the causative agent of Coronavirus Disease 2019 (COVID-19), is highly contagious and significantly threatens public health. This single-stranded positive RNA virus consisting of approximately 30 kb genome size virus is from the same *Sarbercovirus* subgenus as SARS-CoV [1]. While most people who are infected exhibit only mild–moderate respiratory symptoms including cough and dysgeusia, some may develop acute respiratory distress syndrome. Postmortem lungs of COVID-19 patients showed severe pulmonary damage and abundant inflammatory infiltrates [2]. Given the urgent need to study the pathogenesis of this disease and to test the efficacy of potential therapeutics, several in vitro and in vivo models have been developed. Herein, the use and limitations of two-dimensional (2D) and animal models in COVID-19 research are discussed, followed by a review on the use of lung organoids in advancing our knowledge on COVID-19 pathogenesis.

## Two-dimensional models: Their usability in SARS-CoV-2 research and limitations

**Fig 1:**
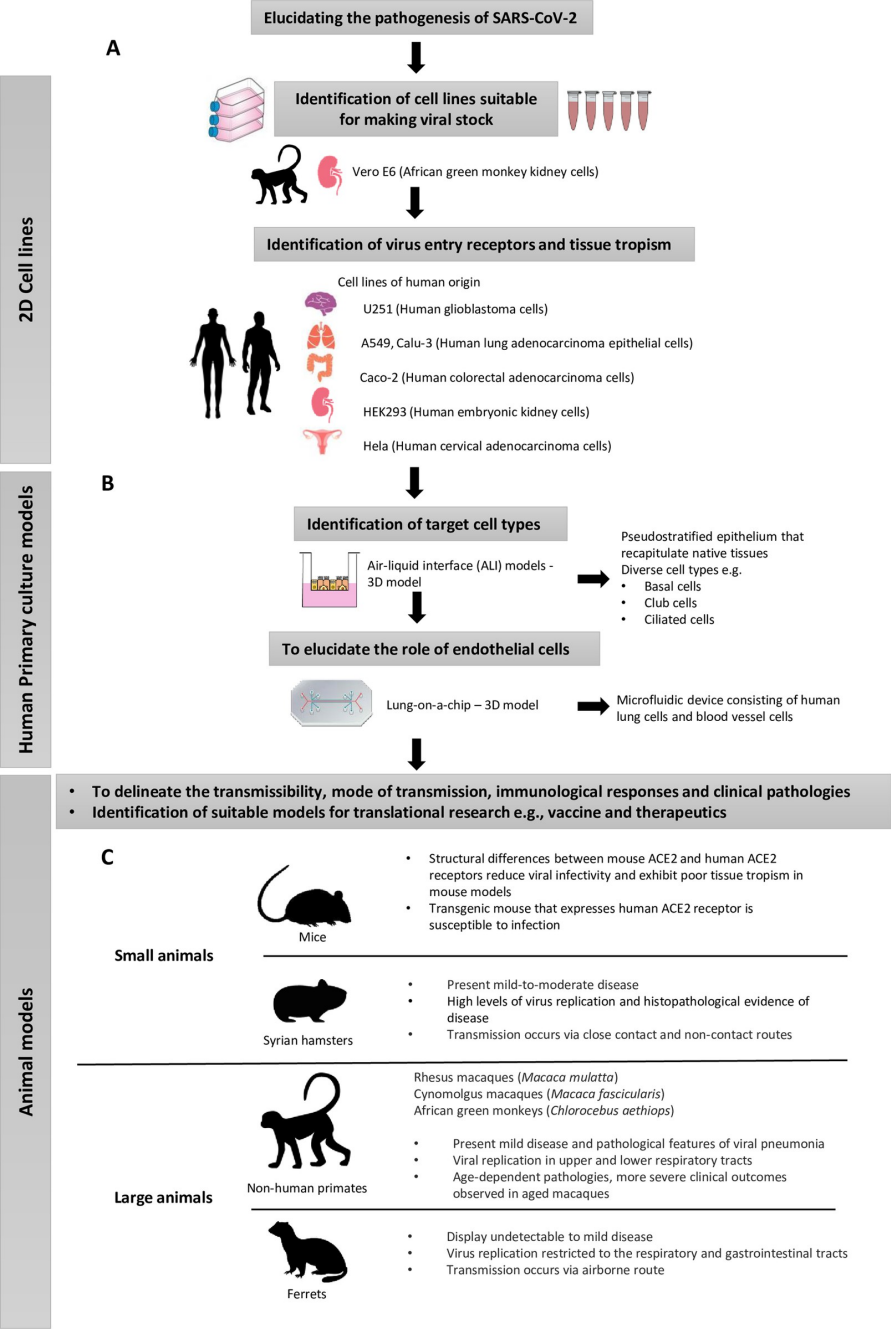
A flowchart illustrates the 2D cell lines, 3D models, and animal models for the understanding of the pathogenesis of SARS-CoV-2. **(A)** Most 2D models originated from both human and nonhuman origins more than half a century ago, thus may not be biological relevant. However, they are easily and readily available, hence form an important first-line model for research in a pandemic to identify potential pathways of infection, tissue tropism and to generate viral stocks for further research. **(B)** Human primary ALI (3D model) mimics conditions in the human airway, as basal surface of cells is submerged in liquid medium, whereas the apical surface is exposed to air. ALI models are useful to identify key target cells of infection and changes in cellular morphologies in infection, whereas more advanced lung-on-a-chip can be applied to understand the role of microvasculature in COVID-19 pathogenesis. **(C)** Animal models are important to identify mechanistic pathways of disease, transmissibility, and preclinical testing of therapeutics. Several models have been tested with varying degree of success. Differences in viral and host homologs render some models incompatible (e.g., wild-type mouse models vs. humanized ACE2 transgenic mouse). Though Syrian hamsters, macaques, and ferrets are susceptible to infection, often they exhibit mild–moderate pathological outcomes. Large animals including macaques and ferrets are protected against reinfection, suggesting that these could be useful models in studying humoral immunity in COVID-19. ACE2, angiotensin-converting enzyme 2; ALI, air–liquid interface; COVID-19; Coronavirus Disease 2019; SARS-CoV-2, Severe Acute Respiratory Syndrome Coronavirus 2; 2D, two-dimensional; 3D, three-dimensional.

Two-dimensional cell lines display a wide range of usability and are readily available in a pandemic for identifying potential pathways of infection and tissue tropism of human pathogens. SARS-CoV-2 cultured in Vero-E6 cells (monkey kidney cells) showed high sequence homology and morphology similarity compared to viruses inoculated in human airway epithelial cells [3,4]. In addition, Vero-E6 cells enable higher level of SARS-CoV-2 amplification and infectivity compared to several human cell lines including A549, Calu-3, HUH7.9, HEK-293, and U251, suggesting its usefulness in generating viral stocks for translational research [5,6]. HEK-293 cells (human embryonic kidney cells) enabled the identification of S1 and S2 subunits of the SARS-CoV-2 transmembrane spike glycoprotein as the ligands that bind host receptors [7]. Using Vero-E6 cells, these subunits were also shown to be involved in viral fusion to host cell membrane to establish infection [8]. Consequently, these cell lines have been used to evaluate drugs and vaccines response [9]. In vitro cell models also enabled the identification of host factors that are involved in SARS-CoV-2 infection. Using parental BHK-21 cells (hamster-derived), the importance of angiotensin-converting enzyme 2 (ACE2) receptor in mediating infection has been discovered [7,10]. Using human HeLa cells, several proteases including transmembrane protease serine 2 and lysosomal protease cathepsin were identified as essential determinants of viral infectivity [7,10]. In addition, genome-wide CRISPR screens in various cell lines have identified functional pathways that are involved in SARS-CoV-2 infection. Screening in Vero-E6 cells led to the discovery of several proviral genes such as HMGB1 (which plays a role in regulating ACE2 expression, hence may affect viral entry into host cells) [11] and CDK4 (which is involved in cell cycle regulation that is crucial for viral replication) [12]. Using the same cell line and technique, the SWI/SNF chromatin remodeling complex has also been identified as proviral, while the Histone H3.3 complex was proposed to have an antiviral role in SARS-CoV-2 infection [11]. While the use of 2D cellular models in research has significantly advanced our understanding on SARS-CoV-2 pathogenesis, there are several noteworthy limitations. These cell lines do not mimic the complexity of the tissue microenvironment and the in vivo conditions in the human primary airway because they are often monocultures of immortalized cells, and some are of animal origins [13,14]. For example, they may not reproduce the antiviral responses in the lungs during an infection. In addition, long-term passaging of SARS-CoV-2 in permissive cell lines may induce mutations that alter the viruses’ original pathogenicity and transmissibility, thus overtime may no longer be usable for research (Fig 1A) [6,15]. Ultimately, reducing capacities to translate novel basic science discoveries to clinical trials, and some of these limitations can be addressed using 3D cultures, which are discussed in later section.

## Animal models: Their usability in SARS-CoV-2 research and limitations

Animal models are important to delineate the transmissibility, immunological responses, and clinical manifestations of respiratory infections. However, physiological differences in the models and the lack of appropriate reagents for some animals may hinder scientific progress. For example, structural differences between mouse and human ACE2 resulted in reduced infectivity in mice because the mouse ACE2 does not support the binding of the original SARS-CoV-2 strain [16]. This limitation was addressed by using transgenic mice expressing human ACE2, but studies showed that disease severities in these mice may differ depending on their age and the inoculation dose, which leads to discrepancies across studies [17,18]. Intriguingly, hamsters are highly susceptible to SARS-CoV-2 infection, as the viruses bind favorably to hamster ACE2 [19]. Infected hamsters showed mild–moderate symptoms and elevated inflammatory responses that mirror clinical pathologies observed in humans, and severe symptoms manifest when the animals were infected with high viral dosage [20–22]. Therefore, hamsters are useful as preclinical models to screen for therapeutic agents; however, the models may be limited by the availability of hamster-specific reagents for assays [23].

Other animal models such as ferrets share similar lung physiologies to humans; hence, it is not surprising that they are naturally susceptible to SARS-CoV-2 and display mild–moderate clinical symptoms [24,25]. Like hamsters, vaccinated or primarily exposed ferrets are fully protected from symptoms upon reinfection, hence are useful as a model of asymptomatic infections or a model to study humoral immunity against SARS-CoV-2 [26–28]. Nonhuman primates are attractive models due to their close phylogenetic and physiological similarities to humans; however, differences in the clinical course of disease have been reported. For example, marmoset is resistant to SARS-CoV-2 infection and does not exhibit clinical symptoms, potentially due to differences in their ACE2 structure [29,30]. In macaques, SARS-CoV-2 inoculation in young rhesus macaques, but not cynomolgus macaques, resulted in moderately severe disease with pulmonary infiltrates and elevated inflammatory mediators [29,31]. Whereas in aged macaques, infection in rhesus macaques showed increased severity of pneumonia that is absent in aged cynomolgus macaques [29,31]. Together, animal models may be more useful in providing mechanistic links in infection as they display a fuller clinical spectrum of respiratory diseases, tissues pathologies, and heightened immune responses; consequently, they may be better models in evaluating vaccine and drug candidates compared to 2D models [32]. However, a degree of differences especially in severe pathological outcomes exist between animal models and humans, which may reduce our ability to fully understand the effectiveness of therapeutics (Fig 1C). In addition, highly trained individuals and high biosafety facilities are required for use of animal models.

## Organoids—A potential powerful model to bridge research gaps

**Fig 2:**
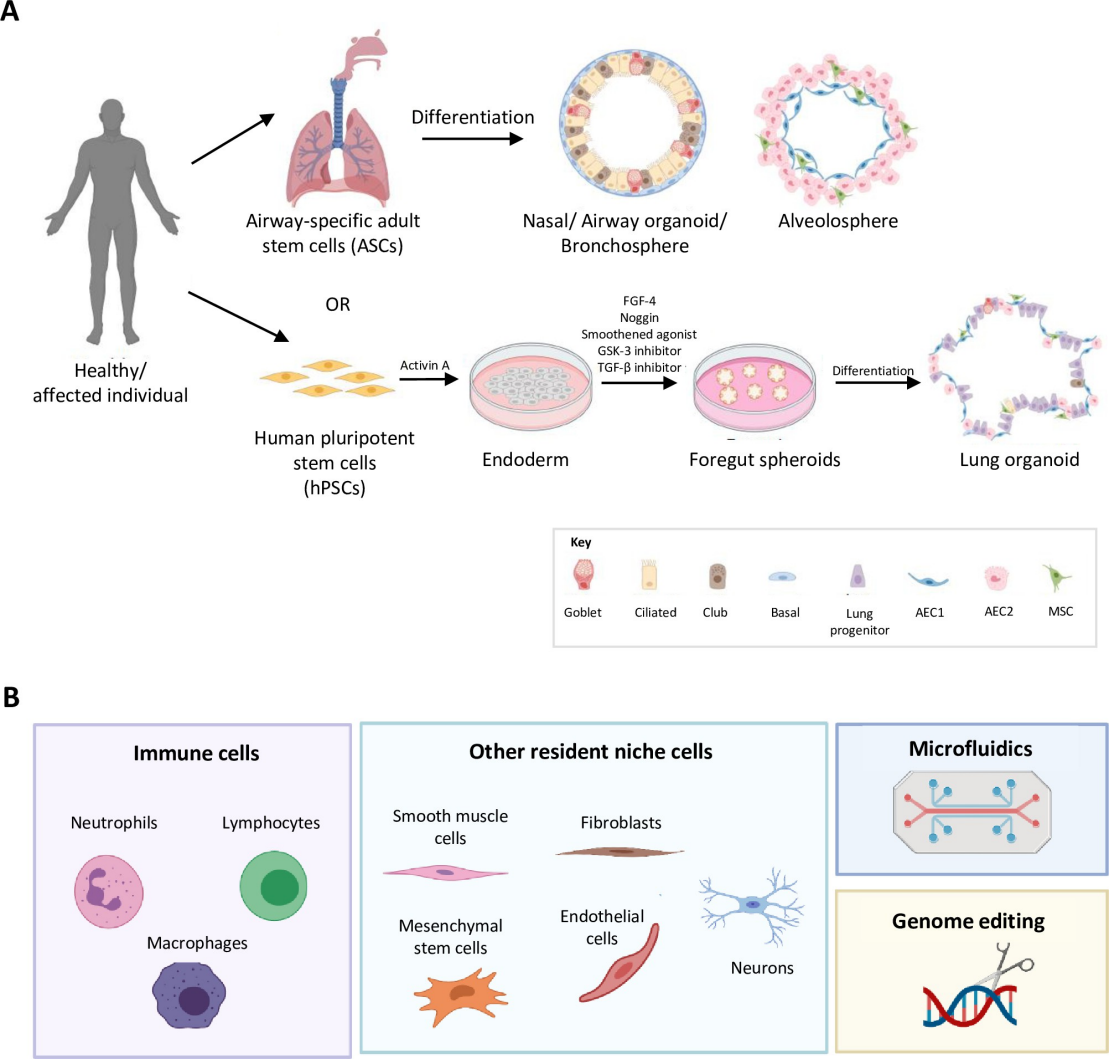
Airway organoids are 3D models that are valuable to bridge the knowledge gap between 2D cell line and animal models. **(A)** Generation of airway organoids from ASCs and hPSCs. ASCs-derived airway organoids (top panel). Tissue-resident adult stem cells can be isolated from healthy individual or patients’ airway samples and differentiated into functional epithelial cell types to form bronchospheres/nasal/airway organoids and alveolospheres with the presence of mesenchymal stem cells. hPSCs-derived lung organoids (bottom panel). Differentiation of hPSCs into endoderm by activin A and then to anterior foregut spheroids through the activation and inhibition of various signaling pathways. Foregut spheroids further differentiate into lung organoids with proximal and distal-like domains that closely resemble the lung morphology and functions. Key: FGF-4 (fibroblast growth factor 4); GSK3 (glycogen synthase kinase 3); TGF-β (transforming growth factor beta 1); AEC1 (alveolar epithelial type I cells); AEC2 (alveolar epithelial type II cells); and MSC (mesenchymal stem cell). **(B)** Future development of next-generation airway organoids. Organoids-on-a chip allows the co-culture of multiple cell types and generation of 3D multicellular structures to better model the functional units of an organ, while replenishment of nutrients and removal of wastes can be done in a controlled manner through microfluidics system. Modification of organoids using advanced gene editing techniques such as CRISPR-Cas9 can be useful for studying virus–host interactions such as deciphering signaling pathways implicated in SARS-CoV-2 pathogenesis or identifying host proteins utilized by viruses in causing infection. Current versions of airway organoids are simplified forms of the native tissue. Incorporation of immune cells and other resident niche cells can highly enhance the biomimicry of the existing airway organoids. Created with BioRender.com. ASC, adult stem cell; hPSC, human pluripotent stem cell; SARS-CoV-2, Severe Acute Respiratory Syndrome Coronavirus 2; 2D, two-dimensional; 3D, three-dimensional.

Organoids are miniature three-dimensional structures, derived either from stem cells that are able to differentiate into organ-specific cell types and self-organize through spatially restricted lineage commitment, or from primary cells through cell sorting to generate structures that recapitulate the functions and architecture of the pertinent tissue [33]. Organoids can be cultured as spheroids cultures or be incorporated as air–liquid interface (ALI) models or lung-on-a-chip models (Figs 1B and 2A). These human airway models have been generated to represent a range of respiratory tissues including the nasal [34,35], bronchial [36], and alveolar regions [37,38] (Fig 2A). Of clinical relevance, these models exhibit epithelial organization and display cellular phenotypes that are representative of the tissues, such as ciliated and alveolar type (AT) 1–2 cells. In vitro¸ airway organoid models provide a valuable platform to study the infectivity of respiratory viruses such as respiratory syncytial virus (RSV) [36], influenza virus [39,40], and SARS-CoV-2 [41–43]. Some disease mechanisms were only modeled in organoids, but not in 2D models. For example, increased cellular motility, an important mechanism for RSV propagation in vivo, was demonstrated in pulmonary organoids but not in traditional 2D models [36]. Furthermore, in ALI models, SARS-CoV-2 showed a gradient infectivity from the proximal to distal respiratory tract, and ciliated cells were identified as the primary cellular target using single cell transcriptomic [43–46]. Similarly, vascularized lung-on-a-chip model has revealed SARS-CoV-2 infection in endothelial cells, causing disruption of barrier integrity and promoting a pro-coagulatory microenvironment [47]. However, the generation cost is high, especially for lung-on-a-chip models, which are rarely applicable in large-scale studies. Nevertheless, organoids have a few advantages over standard cell lines and animal models, where they require fewer ethics considerations compared to animal models and are better representation of human tissues compared to 2D cell lines.

## Airway organoids as tools to investigate pathogenesis in SARS-CoV-2

The use of human stem cell–derived airway organoids has proven to be extremely useful in SARS-Cov-2 research, as they have led to observations that are difficult to delineate and validate using either 2D cell lines or animal models [37,48,49]. For example, studies using 2D cell lines showed distinctive modes of entry by SARS-CoV-2 into host cells; this uncertainty in the viruses’ mechanism of entry hinders the process of designing specific inhibitors [7,10]. Using adult bronchial-derived organoid, it was confirmed that serine proteases, but not cathepsins, were crucial in mediating virus entry into airway organoids [7,50]. Alveolar organoids were shown to be more resistant to SARS-CoV-2 infection compared with bronchial organoids, possibly due to their lower levels of ACE2 expression; hence, SARS-CoV-2 demonstrates tissue tropism toward the bronchial region through microaerosol inhalation [43,48]. Furthermore, viruses propagated in bronchial and bronchiolar organoids displayed phenotypic and genetic similarities with those from SARS-CoV-2 patients; they were also less likely to induce mutations, which is common in 2D models [51]. In airway organoids infected with the SARS-CoV-2 alpha variant, higher amount of infectious virus was produced compared to its ancestry strain, which may explain the higher transmissibility of this variant strain [52]. Furthermore, studies with infected AT-2 organoids demonstrated increased genes expression of type 1/3 interferons, chemokines, and apoptosis signals, highlighting organoids as possible models to study tissue-specific pathologies [37,42].

## Advancement in airway organoid models and future directions

While the airway organoid model systems hold great promise in respiratory infection research, there is still room for improvement for existing models. The biomimicry of available organoids is still far from achieving the complexity of its native tissue. Although current versions of airway organoids present multiple differentiated epithelial cell types within the lungs, they are not representative of the systemic response during an infection. Hence, successful incorporation of nonepithelial cells such as immune, stromal, and vascular cells, as well as noncellular components, is necessary to advance existing models. For instance, coculture of alveolar macrophages with human-derived lung cells and SARS-CoV-2 demonstrated elevated levels of proinflammatory cytokines that are likely to contribute to cytokine storm in severe COVID-19 patients [53,54]; hence, a coculture system could better recapitulate the host inflammatory responses induced in vivo. Enrichment of airway organoids with lung endothelial cells is crucial to understand endothelial cell infection and endotheliitis that were shown to contribute to life-threatening COVID-19 complications including venous multiple organ failure and thromboembolic disease [55,56]. In addition, organoids also possess a demonstrable capacity for genome editing. CRISPR-Cas base editing can be applied to decipher signaling pathways implicated in SARS-CoV-2 pathogenesis. Moreover, advances in microfluidic technologies now allow organoid engineering at an unprecedented scale, permitting essential structural and physiological characteristics to be maintained in a sophisticated and controlled manner, hence minimizing batch-to-batch variation with greater data consistency and amenable for large-scale productions (Fig 2B) [57].

## Conclusions

COVID-19 has fast-forwarded the applications of airway organoids in respiratory infection research including cellular and inflammatory responses and therapeutics screening [41]. Airway organoids present numerous advantages over the standard 2D cell lines and primary airway cultures, as they comprise multiple cell types that can be more representative of in vivo conditions, without the complexities involved in using animal models. Lastly, they are readily accessible to study any new SARS-CoV-2 variants and emerging infectious diseases for pandemic preparedness and treatment intervention.
